# Adipose Tissue-Resident Immune Cells in Obesity and Type 2 Diabetes

**DOI:** 10.3389/fimmu.2019.01173

**Published:** 2019-05-22

**Authors:** Jingli Lu, Junjie Zhao, Haiyang Meng, Xiaojian Zhang

**Affiliations:** ^1^Department of Pharmacy, the First Affiliated Hospital of Zhengzhou University, Zhengzhou, China; ^2^Henan Key Laboratory of Precision Clinical Pharmacy, Zhengzhou University, Zhengzhou, China

**Keywords:** obesity, type 2 diabetes, inflammatory responses, adipose tissue-resident immune cells, immunometabolism, immunotherapy

## Abstract

Inflammation is an important contributor to the pathogenesis of obesity-related type 2 diabetes (T2D). Adipose tissue-resident immune cells have been observed, and the potential contribution of these cells to metabolic dysfunction has been appreciated in recent years. This review focused on adipose tissue-resident immune cells that are dysregulated in the context of obesity and T2D. We comprehensively overviewed emerging knowledge regarding the phenotypic and functional properties of these cells and local factors that control their development. We discussed their function in controlling the immune response cascade and disease progression. We also characterized the metabolic profiles of these cells to explain the functional consequences in obese adipose tissues. Finally, we discussed the potential therapeutic targeting of adipose tissue-resident immune cells with the aim of addressing novel therapeutic approaches for the treatment of this disease.

## Introduction

Type 2 diabetes (T2D), which is characterized by insulin resistance and continuous islet β cell dysfunction, is a highly heterogeneous and chronic disease (1). Although genetic backgrounds and epigenetic factors both pose risks for T2D development, obesity-induced inflammation is an important mechanism in the pathogenesis of T2D (2). According to the novel diabetes classification of five subgroups of adult-onset diabetes, obesity is a crucial feature in two clusters of patients with diabetes, including mild obesity-related diabetes and insulin-resistant diabetes with severe obesity (3).

Obesity is accompanied by a chronic inflammation of adipose tissue, and this inflammation impairs glucose metabolism (4). Studies in animal models, particularly in adipose tissue-restricted Cre mice, have identified roles for several different types of immune cells residing in adipose tissue. Adipose tissue-resident immune cells, including T cells (5), B cells (6), macrophages (7), and dendritic cell (DC) subsets (8, 9), are dysregulated in the context of obesity and are associated with the development of a disturbed immune system and progression of the disease. Other unconventional lymphocyte subtypes that reside in adipose tissue also contribute to tissue homeostasis and the control of metabolic function. These cells include invariant natural killer T (iNKT) cells (10), mucosal-associated invariant T (MAIT) cells (11), γδ T cells (12) and innate lymphoid cells (ILCs) (13). Therefore, it is not surprising that targeting some molecules that can control the activity of immune cells, such as interleukin (IL)-1 (14) and tumor necrosis factor (TNF) α (15), has been reported to be effective in diminishing diabetes progression.

This review focuses on dysfunctional immune cells that reside in the adipose microenvironment in the context of obesity and T2D. First, the general role of adipose-resident immune cells was summarized. We reviewed their phenotypic and functional properties in the context of obesity and T2D and summarized additional modulators that control their migration, survival, proliferation, and effector functions. The focus of the second section was on metabolic regulation that determined the functional states of immune cells residing in adipose tissues. Metabolic changes in these cells also contributed to the pathogenesis of obesity-related T2D. The third section discussed the potential therapeutic targeting of adipose tissue-resident immune cell dysfunction with the aim of further promoting progress in the development of new therapeutic approaches for T2D.

## The Role of Adipose Tissue-Resident Immune Cells in Obesity and T2D

**Figure 1:**
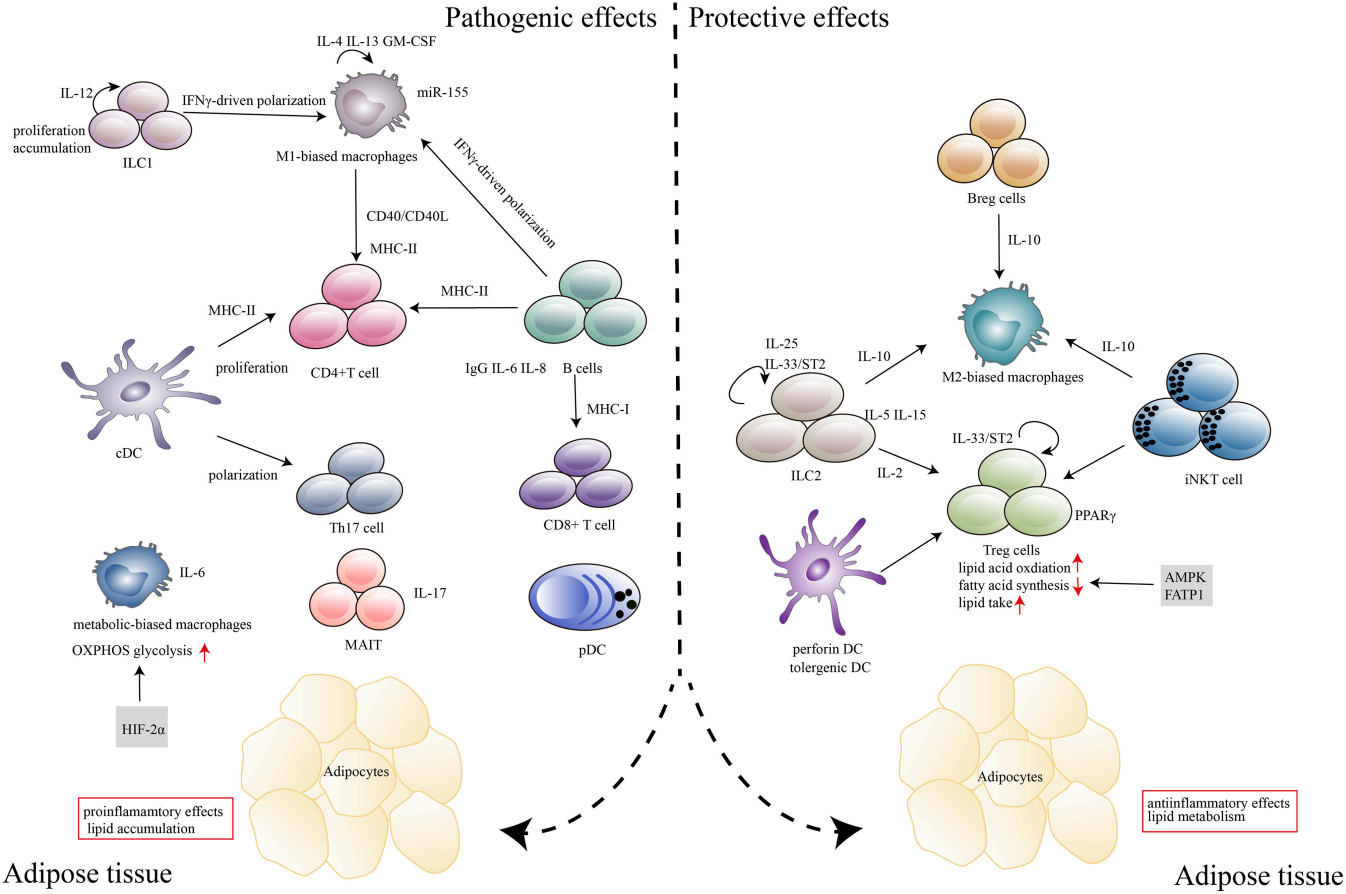
Immune cells in adipose tissues in the context of obesity and T2D. In response to excess nutrients, low levels of inflammatory mediators, such as TNF-α, CCL2, and IL-1β, are produced, which induces the recruitment and activation of immune cells in adipose tissue. Major histocompatibility complex (MHC) molecules expressed by antigen-presenting cells, including DCs, macrophages and B cells, induce CD4^+^T, and CD8^+^ T cell expansion. Cytokines (IL-6, IL-8, IL-17, and IFN-γ) that are secreted by immune cells (B cells, Th17, MAIT, and M1 macrophages) can polarize, proliferate and accumulate other inflammatory cells in adipose tissues. IgG produced by B cells also plays a deleterious role in disease development. Metabolism-related molecules such as HIF-2α, AMPK, and FATP1 control immune cell differentiation and function, which contribute to the pathogenesis of obesity and T2D. The marked infiltration of adipose tissues by immunosuppressive cells, including Treg cells, Breg cells, ILC2s, M2 macrophages, tolerogenic DCs and iNKT cells, which abundantly produce anti-inflammatory cytokines (IL-10, IL-15, IL-2, IL-5, and IL-25) within the adipose microenvironment, contributes to the protective effects in disease progression.

Inflammation occurs as a consequence of chronic nutrient excess in obesity, which induces insulin resistance and impaired glucose metabolism. As the field has matured, it has revealed that this process involves a wide range of components and is regulated by a complex network. First, intracellular lipids, such as diacylglycerols and sphingolipids, can serve as lipotoxic substances by directly inhibiting insulin activity in muscles and the liver (16). Second, much of the early studies focused on how inflammatory cytokines and chemokines, such as TNFα, CCL2, IL-6, IL-4 and IL-1β, contribute to T2D (17, 18). Clinical studies using biological agents that target individual cytokines, such as TNF antagonism and IL-1β antagonism, to improve glucose metabolism are the most relevant in this regard (19). Third, the activation of key inflammatory mediators, such as c-Jun N-terminal kinase (JNK) and IκB kinase (IKK), also contributes to obesity-associated insulin resistance and deterioration of glucose metabolism (20). More recently, however, attention has shifted to the infiltration of immune cells, especially those subtypes with distinct phenotypes and effector functions, into adipose tissues, and this is the main focus of this review (Figure 1).

### Macrophages

Over 50% of immune cells in obese adipose tissues are macrophages, while fewer than 10% of immune cells in lean adipose tissue are macrophages (21–23). Macrophages in subcutaneous and visceral adipose tissue correlated with total adiposity and adipose cell size, and secreted the majority of inflammatory cytokines in response to obesity (21–23). Macrophage infiltration into the adipose tissue of obese mice switches the macrophage phenotype from alternatively activated (M2) polarization with canonical markers (Arg1, CD206, and CD301) toward the proinflammatory (M1) phenotype with the expression of Nos2, Tnfa and Itgax (24–26), which can directly impair insulin activity (21, 27–29). Additionally, a more complex immunophenotype of macrophages that does not conform to the traditional macrophage activation archetype of M1- and M2-biased populations has also been observed in obese adipose tissue (30). These cells have a unique metabolically activated macrophage phenotype, which can be induced by external stimuli with mixtures of glucose, insulin, and palmitate-conditions characteristic of metabolic syndrome, with elevated metabolic markers (*Plin2* and *Abca1*) and proinflammatory cytokines (*Tnf*α and *Il*β) (30). Analysis of macrophages from obese subjects produced similar findings that macrophage metabolic activation correlated with adiposity (30).

Consistent with the macrophage phenotype, recently published data have described the unexpected role of macrophages in the development of obesity and T2D in a manner that is independent of inflammatory activation. Macrophages can clear dead adipocytes that induce the expression of *IL1*β and *IL6*, so their clearance was associated with the inhibition of inflammation in obesity (31). Since macrophages are much smaller than adipocytes, they clear dead adipoctyes through lysosomal exocytosis, which can be driven by NADPH oxidase 2 (31). Inhibition of macrophage lysosome function impairs lipid metabolism and increases lipid content in macrophages and reduces whole adipocyte lipolysis (32). Macrophage exosomes from obese adipose tissues also contain miR-155, which targets peroxisome proliferator-activated receptor (PPAR) γ and can cause insulin resistance (33). These data argue that macrophages not only contribute to the development of obesity-induced inflammation but also serve an important role in lipid metabolism independent of their inflammatory phenotype.

A major tissue-resident macrophage population is established prior to birth (34), but further proliferation of macrophages residing in adipose tissues likely occurs in the context of obesity-induced chronic inflammation. Adipose tissue macrophages are poised for proliferation by cytokines IL-4, IL-13 and granulocyte-macrophage colony stimulating factor (GM-CSF), whereas TNF-α inhibits their proliferation (35). CD11b deficiency promotes *in situ* adipose tissue macrophage proliferation, which is a process mediated by the IL-4/STAT6 signaling pathway (36). It is conceivable that additional immune cells might be similarly affected by these cytokines; therefore, specific factors that control macrophage proliferation in obese adipose tissues need to be investigated.

Much is known about molecules that regulate the polarization of macrophages in obesity. First, macrophage polarization is influenced by endoplasmic reticulum (ER) stress. Indeed, the transcription factors C/EBP homologous protein (CHOP) and inositol-requiring enzyme 1 (IRE1) are important components of ER stress pathways and are essential in the induction of M1 polarization of infiltrating macrophages during obesity development (37, 38). Second, epigenetic alterations determine macrophage polarization. Corepressor complexes containing G protein pathway suppressor 2 (GPS2) cooperate with silencing mediator of retinoid and thyroid hormone receptors to repress proinflammatory gene expression (39). Sirtuin 6 (Sirt6) deletion in macrophages promotes positive feedback circuits for nuclear factor-κB (NF-κB) and STAT3 activation stimulation, which expedites M1 polarization (40). Third, several data have suggested the potential for inflammatory pathways to affect macrophage function in obesity. JNK in macrophages is required for inflammatory macrophage polarization, which contributes to the establishment of obesity-induced insulin resistance and inflammation (41). PB1 domain-containing adaptor NBR1 and mitogen-activated kinase kinase 3 (MEKK3) signaling complex formation and mammalian target of rapamycin complex 1 (mTORC1) signaling could trigger JNK-mediated inflammation and insulin resistance (42, 43). Finally, transcription factors control the polarization of macrophages in obesity. It has been described that inflammatory macrophages expressing interferon regulatory factor 5 (Irf5) accumulate in obese adipose tissue and that Irf5-deficient macrophages selectively induce adipose tissue remodeling (44). These data show that multiple types of mechanisms may function in macrophage polarization, but the mechanism that is central in this process remains to be determined.

Adipocytes and immune cells act cooperatively to produce cytokines and chemokines that mobilize the rapid recruitment of inflammatory macrophages. For example, CXCL12 (45), CCR5 (46) and semaphorin 3E (47, 48) promote the infiltration and recruitment of macrophages, whereas CX3CR1 (49) is not required for the recruitment or retention of macrophages in obese adipose tissues. IL-6 signaling plays an important role in alternative activation and recruitment of macrophages and metabolic homeostasis (50); this cytokine is constantly produced in obesity. Interestingly, selectively blocking IL-6 trans-signaling, unlike complete ablation of IL-6 signaling, does not exacerbate obesity-induced weight gain or insulin resistance (51). In addition, macrophages can be retained by macrophage-adipocyte adhesion, and this interaction directly inhibits beige adipogenesis (52). Intricacies have also been observed in studies of macrophage infiltration into adipose tissues. Although a large body of evidence supports that the CCL2/CCR2 system increases macrophage infiltration into adipose tissue (53–56), other studies have reported conflicting results showing that disruption of CCL2 or CCR2 expression does not decrease the activity of macrophages (57, 58). Molecular mechanisms underlying immune mediators that control macrophage infiltration into adipose tissue require further clarification, particularly because these conflicting findings underscore the pleiotropic functions of single molecules.

Overall, it seems that macrophages are an important component in adipose tissues that are dedicated to controlling pathological processes via immune and non-immune functions in obesity. Multiple types of regulatory mechanisms may be independently involved in the proliferation, recruitment, and polarization of macrophages in obese adipose tissues, but most of them have not been characterized with regard to tissue-specific importance for macrophage function.

### Dendritic Cells (DCs)

Early studies described the presence of conventional DCs (cDCs) in adipose tissues (24, 59, 60), and depletion of these cells has been shown to result in a rapid normalization of insulin sensitivity and a decrease in proinflammatory cytokines in obese mice (8, 9), suggesting a pathogenic role for cDCs in obesity. High-fat diet-derived DCs are skewed toward a proinflammatory phenotype with increased IL-1β secretion and expression of IL-1R1, toll-like receptor (TLR) 4, and caspase-1 (61). cDC accumulation during obesity has been shown to be attenuated in Ccr7^−/−^ mice and associated with decreased adipose tissue inflammation and lowered fasting glucose and insulin levels, suggesting that DCs are only partially CCR7-dependent (62, 63). In humans, cDCs were also identified and accumulated in obese adipose tissue that expressed CCR7 (62, 63). Unlike cDCs, plasmacytoid DCs (pDCs) have a decreased capacity to take up, process and present soluble antigens. At least two independent studies noted that pDC accumulation in adipose tissues can be detrimental to obesity (64, 65). In one study, the development of obesity and insulin resistance in this model was shown to be associated with pDC recruitment to adipose tissue, and depletion of pDCs protected the mice from diet-induced obesity and obesity-associated metabolic complications (64). In obese individuals, adipose-recruited pDCs have been shown to be associated with deregulation of a specific adipokine (65). These studies suggest a pathogenic role for cDCs/pDCs in the development of obesity in mice and humans.

A contradiction to this hypothesis is that a rare subpopulation of cDCs, perforin^+^ DCs, play a protective role in obesity. These results were obtained with a bone marrow transplantation model that selectively lacked perforin^+^ DCs (66). The data demonstrated that mice with perforin^+^ DC deficiency progressively gained weight and exhibited features of metabolic syndrome (66). This phenotype was shown to be associated with an altered repertoire of T cells residing in adipose tissue and could be completely prevented by T cell depletion *in vivo* (66), suggesting a DC-T cell interaction in the obesity-associated syndrome. Whether specific adipose tissue DC signature may exist in human, is currently known. Interestingly, cDCs in visceral adipose tissue could acquire a tolerogenic phenotype by β-catenin and PPARγ activation (67). cDC-specific deletion of β-catenin and PPARγ did not affect weight gain, visceral adipose tissue content, or food intake, but enhanced local inflammatory responses and aggravated obesity-induced insulin resistance (67). The results suggest that the tolerogenic properties of adipose tissue DCs may serve as a checkpoint for the control of tissue inflammation. Nevertheless, it is likely that DCs can show phenotypical and functional plasticity in the context of obesity, which provides a foundation for further investigation to define the precise role of DCs in obesity and T2D.

### Lymphocytes

Lymphocytes and their subpopulations that control the development of obesity are being identified at a rapid pace, building on our knowledge of adipose tissue-resident lymphocytes in obesity-related T2D. The development of insulin resistance in RAG1-deficient mice showed a clear role for lymphocytes against the deleterious effects of obesity (68).

At steady state, T cells compose ~5–10% of the hematopoietic compartment in visceral and subcutaneous adipose tissue, which is a major hub for memory T cells with potent proliferative and effector potential (69). In humans, obesity increased the frequency of CD4 and CD8 cells in adipose tissue (70). Studies in obese mice have shown that T cells generally accumulate in obese adipose tissue (71–73). The ability of short-term depletion of T cells in fat by anti-CD3 to reverse insulin resistance in early-stage obesity emphasizes the key role of adipose T cells in improving glucose tolerance and insulin sensitivity (68, 70). An increase in the ratio of CD8^+^ to CD4^+^ adipose tissue T cells has been observed, although each study addressed different T cell subsets in this process (68, 71, 74, 75). In general, CD8^+^ T cells and proinflammatory T helper 1 (Th1) cells have a pathological role, whereas anti-inflammatory Th2 cells play a protective role in obesity and T2D (74–76). In particular, insulin resistance development depends on CD8^+^ effector T cells as follows: CD8^+^ T cell infiltration into adipose tissues precedes the accumulation of macrophages; genetic depletion of CD8^+^ T cells decreases adipose tissue inflammation and ameliorates systemic insulin resistance; and adoptive transfer of CD8^+^ T cells to CD8-deficient mice aggravates adipose inflammation (71).

However, the discovery that a small subpopulation of T cells in adipose tissues with a unique phenotype has effects on the inflammatory response adds to the complexity of how T cells regulate obesity development. For example, a unique subpopulation of CD153^+^PD-1^+^CD44^hi^CD4^+^ T cells that express T-bet with negligible GATA3, RORγ, and Foxp3 expression has been shown to increase and accumulate in obese adipose tissues and to cause adipose tissue inflammation and systemic insulin resistance in high-fat diet-fed mice (77). Functionally, CD153^+^PD-1^+^CD44^hi^CD4^+^ T cells were shown to produce large amounts of osteopontin upon T cell receptor (TCR) stimulation, whereas the production of IFN-γ and IL-2 was shown to decrease in CD153^+^PD-1^+^CD44^hi^CD4^+^ T cells compared with PD-1^−^CD4^+^ T cells (77).

Regulatory T (Treg) cells contribute substantially to the control of the inflammatory state of adipose tissue because they are highly enriched in the fat of normal mice, but their numbers have been shown to be strikingly and specifically reduced at this site in obesity (74, 78, 79). In humans, Foxp3 mRNA was detectable in fat depots, which was correlated with BMI (74). Fat Treg cells have a unique signature with several chemokine and chemokine receptors (Ccr1, Ccr3, Cxcr6, Cxcl2, Ccr2, and Ccr9), immunomodulatory cytokines (such as IL-10), transcription factors (Pparg, Gata3 and Irf4) and encoded molecules involved in lipid metabolism (Pcyt1a, Dgat1, Ucp1, Lipe, and Plin1), all of which have unique functional properties that locally control metabolic indices (74, 78, 80–82). Fat Treg cells have been seeded from thymocytes and were shown to accumulate depending on the expression of TCR, Foxp3, and IL-33 receptor (ST2) in the local microenvironment (83, 84). TCR crosslinking induces PPAR-γ and ST2 expression, and MyD88-mediated signaling downstream of IL-33 further upregulates ST2 expression and expands fat Treg cells (79). The AP-1 transcriptional regulator BATF and the transcription factor IRF4 are necessary for fat Treg differentiation through direct regulation of ST2 and PPAR-γ expression (79). Fat Treg cells are important for the maintenance of immune homeostasis and improve glucose homeostasis in obese mice.

T cells are clearly pivotal for T2D development, but there are also data suggesting an involvement of B cells. B cell depletion in diet-fed mice, either through targeting particular genes or antibody treatments, has been shown to improve glucose tolerance and reduce adipose tissue inflammation (6, 85). B cells from obese mice secrete more proinflammatory (IFN-γ, IL-6, and IL-8) and less anti-inflammatory (IL-5 and IL-10) cytokines (6). B cells can also exert their detrimental effects through the production of pathogenic IgG antibodies (6). Although total B cells may have a deleterious role in disease development, adipose tissue regulatory B (Breg) cells are positively associated with insulin sensitivity and restrain adipose tissue inflammation (86). Breg cell function and survival support adipose environmental factors, such as IL-10, CXCL12 and free fatty acids (FFAs) (86). The role of adipose tissue B cells in obesity and T2D has not demonstrated.

Therefore, our current knowledge indicates that the inflammatory response during obesity is coupled with activation/inactivation or changes in the numbers of lymphocyte populations. As described above, there is compelling evidence for heterogeneity among lymphocytes in adipose tissues, which may have compensatory roles in maintaining adipose immune homeostasis. However, the relative contribution of these lymphocytes in obesity remains incompletely understood.

### Unconventional Lymphocytes

In contrast to the classic view of adaptive lymphocytes in obesity and T2D, studies in the past decade have led to the characterization of unconventional T cells, such as invariant natural killer T (iNKT) cells, mucosal-associated invariant T (MAIT) cells, γδ T cells, and the emerging family of ILCs.

#### iNKT Cells

iNKT cells, which are significantly enriched in adipose tissues of both mice and humans, are innate-like T cells that have a semi-invariant αβTCR and represent 15–20% of total T cells (10, 87). Mice lacking iNKT cells have been shown to have enhanced weight gain, larger adipocytes, fatty livers, and insulin resistance on a high-fat diet (10). Increasing the frequency of iNKT cells, either by adoptive transfer or *in vivo* activation of iNKT cells, substantially improves the metabolic outcome and weight loss (10, 88–90). Moreover, a study described a correlation between the frequencies of iNKT cells and excessive visceral fat accumulation in lean/obese individuals (91). Early reports suggested that an iNKT cell-mediated improvement in insulin sensitivity is associated with Th2 cell-type cytokine production by adipose-derived iNKT cells (10).

Notably, adipose iNKT cells have a unique transcriptional program with the overexpression of the MAP kinase phosphatase Dusp1 and the nuclear receptor transcription factor Nur77 (Nr4a1) (92). Unlike hepatic, splenic and thymic iNKT cells, adipose iNKT cells lack the poxvirus zinc finger transcription factor PLZF but express the basic leucine zipper transcription factor E4BP4 (Nfil3), which controls the production of IL-10 (92). The enrichment of regulatory iNKT cells in adipose tissue maintains inflammation in a quiescent state and regulates the homeostasis of other anti-inflammatory immune cells, including M2 macrophages and Treg cells (92). However, not all studies have suggested a protective role of iNKT in obesity, possibly due to the lower TCR diversity of the Traj18^−/−^ mice used in the study (93). Therefore, a more complete understanding of the detailed TCR repertoire in controlling iNKT cell development and how these cells respond to TCR activation need to be elucidated and could provide new insights into the effector function of iNKT cells in obesity.

#### MAIT Cells

MAIT cells are a novel subset of innate-like immune cells expressing an invariant TCR α chain (dVα19Jα33 in mice and Vα7.2-Jα33 chain in humans) (94). MAIT cells are more prone to accumulate in adipose tissues and exhibit a striking IL-17 profile in patients with obesity (11, 95). Although the alterations in MAIT cells might contribute to obesity-related inflammation, no detailed insights exist as to how MAIT cells develop and function in adipose inflammation in the disease context by using mouse studies with genetic removal or activation of MAIT cells.

#### γδ T Cells

γδ T cells constitute another subset of unconventional T cells representing 1–10% of circulating T cells in humans and mice. A recent study has shown that γδ T cells are decreased in the circulation of obese subjects and negatively correlated with body mass index (12). γδ T cells were found to be enriched and resident in mouse and human adipose tissues (96). γδ T cells in adipose tissue could regulate age-dependent Treg cell expansion (96). The potential protective role of γδ T cells in obesity is further supported by the fact that γδ T cells could impact metabolic processes by suppressing differentiation of the 3T3-L1 preadipocyte cell line and impairing glucose uptake by mature 3T3-L1 adipocytes (97). Notably, γδ T cells preferentially accumulated in inguinal adipose tissue, the colon and small intestine lamina propria during obesity (98–100). The above studies implied that γδ T cells might exert immune and metabolic functions depending on the tissue microenvironment.

#### Innate Lymphoid Cells (ILCs)

ILCs as part of the innate immune system are characterized by classic lymphoid cell morphology, but unlike other immune cells, ILCs are defined on the basis of their expression of effector cytokines and other functional molecules (101). Recent data highlighted the role of ILCs in response to nutrient and metabolic stress in obesity and T2D. A population of IL-5- and IL-13-producing ILC2s in murine white adipose tissue maintains eosinophil and alternatively activated macrophage responses that limit high-fat diet-induced obesity and insulin resistance (90, 102). In human, adipose tissue from obese subjects exhibited decreased frequencies of ILC2s compared to non-obese controls (13). Adipose tissue ILC2s were developmentally dependent on inhibitors of DNA binding 2 (Id2), transcription factor 7 (TCF-7) and the common gamma chain (cc) and produced enkephalin peptides, which is a previously unrecognized effector mechanism employed by ILC2s to regulate metabolic homeostasis (13). Consistent with this, IL-25-elicited ILC2 responses were associated with lower weight gain following exposure to a high-fat diet (90), suggesting that ILC2 responses may play an important role in regulating adipocyte development and/or function in the context of obesity. In contrast, IFNγ-producing ILC1s that selectively accumulate in adipose depots contribute to insulin resistance following diet-induced obesity (103). The deleterious role of adipose tissue-resident ILC1s has been further highlighted in a recent study that showed that obesity contributed to loss of cytotoxic function of ILC1s, depletion of ILC1s resulted in alterations in the ratio of inflammatory to anti-inflammatory macrophages, and adoptive transfer of ILC1s exacerbated metabolic disorder (104). Thus, the ILC1 and ILC2 subsets in adipose tissues exhibit remarkable differences in terms of cytokine expression and effector function in obesity.

## Metabolic Regulation of Adipose Tissue Immune Cells

Emerging evidence indicates that intracellular metabolic changes not only codetermine immune cell differentiation and function but also contribute to the pathogenesis of obesity and T2D. Next, we focused on macrophages and Treg cells, for which metabolic mechanisms have begun to be uncovered in obesity and T2D. Functional consequences of metabolic programming in these cells are expected to lead to novel therapeutic targets that are polarized toward their particular effector functions that ultimately prevents disease progression.

### Macrophages

It has been shown that mice lacking PPARγ (105, 106), PPARδ (107, 108) and PPARγ coactivator-1β (PGC1β) (109), which are key regulators of lipid oxidation and mitochondrial biogenesis, are prone to lipid-induced inflammation and insulin resistance. Accordingly, myeloid-specific deletion of PPARγ exacerbates macrophage inflammation and promotes the development of diet-induced obesity (106). In these studies, a close association between metabolism and macrophage function in obesity was shown. Obesity has been shown to induce lysosome biogenesis in adipose macrophages, and inhibition of lysosome function has been shown to impair lipid metabolism in these cells and reduce whole adipose tissue lipolysis (32). In this role, increased lipid content and altered lipid metabolism in adipose tissue-resident macrophages is associated with low-grade inflammation in obesity (32).

A key signaling pathway that controls macrophage lipid metabolism during the course of obesity is regulated by the metabolic sensor adenosine monophosphate (AMP) kinase (AMPK) (110). AMPK antagonizes biosynthetic pathways; for example, by increasing fatty acid oxidation and suppressing fatty acid synthesis through the phosphorylation of acetyl-CoA carboxylase (ACC) (111). AMPK has also been shown to promote catabolic processes through regulation of total mitochondrial content, which is mediated by the activation of PGC1α (112). In macrophages, a deficiency in AMPKβ1 increases the levels of diacylglycerol and markers of inflammation and suppresses the expression of mitochondrial enzymes and ACC phosphorylation, which results in increased macrophage lipid accumulation (110). When challenged with a high-fat diet, mice that received AMPKβ1^−/−^ bone marrow showed enhanced adipose tissue macrophage inflammation and insulin resistance (110). This result indicates that AMPK signaling plays an important role in the metabolic control of adipose tissue macrophages.

Notably, adipose tissue-resident macrophages exhibit an exclusively metabolically activated phenotype in obesity (30, 32). Saturated FFAs, such as palmitate, initiate signal transduction pathways that mediate lipid metabolism and inflammation in macrophages. Palmitate binds to cell surface TLRs in which proinflammatory cytokine production and palmitate are internalized by macrophages that activate p62 and PPARγ, thereby promoting the metabolic phenotype of macrophages (30). Consistent with the role of fatty acid metabolism in regulating macrophage activation, fatty acid transport protein 1 (FATP1) modulates lipid mediators and oxidative stress to reduce macrophage infiltration and inflammation in adipose tissue (113). Conversely, the fatty acid translocase and scavenger receptor CD36 does not appear to affect saturated fatty acid-induced macrophage lipid accumulation (114).

However, recent reports have revealed the crucial role of glycolysis in obese adipose tissue macrophages. Based on a recent study of macrophage transcriptomes, obese adipose tissue-resident macrophages adopt specific metabolic programs characterized by the activation of various metabolic routes, including both oxidative phosphorylation and glycolysis (7). In particular, glycolysis contributes to increased inflammatory cytokine production, and inhibition of glycolysis with 2-DG reduces both lactate production and glucose oxidation by macrophages in obese adipose tissue (7). When adipose tissue expands enough to produce hypoxia, hypoxia-inducible factor (HIF)-1 is classically activated to promote glycolysis by inducing the expression of enzymes in the glycolysis pathway. However, recent evidence has suggested that adipose tissue inflammation does not require HIF1α-mediated signaling (7) but instead relies on HIF2α (115). Targeting the inhibition of HIF2α markedly augments palmitate-induced proinflammatory gene expression in adipocytes, and HIF2α deficiency in high-fat diet-fed mice exacerbates adipose tissue inflammation (115).

Therefore, these studies suggest a close functional link between fatty acid oxidation, glycolysis, and the metabolic phenotype of macrophages in adipose tissues that play an important role in diet-induced inflammation and obesity. These studies also suggest the possibility that the function of adipose tissue-resident macrophages in obesity is determined by metabolic pathways. It has been described that fatty acid synthase deficiency in macrophages prevents their recruitment in adipose tissue and is associated with impaired retention of plasma membrane cholesterol and disruption of Rho GTPase trafficking, which is a process required for cell adhesion, migration and activation (116).

### Treg Cells

Treg cells residing in adipose tissue with a nutrient-rich environment can take up lipids, and this distinguished property is not shared by Treg cells residing in lymphoid tissue or by conventional T cells residing in nutrient-rich adipose tissue (78). PPARγ-driven fatty acid metabolism is an important factor that controls the phenotype of adipose Treg cells, showing a PPARγ-dependent enrichment of cells expressing GATA3 and Foxp3 (78, 82). Consistent with this observation, treatment with the thiazolidinedione (TZD) drug pioglitazone, which is a synthetic agonist of PPARγ, leads to augmentation of a set of lipid metabolism genes, including those coding for fatty acid transporters (Cd36 and Slc27a2), enzymes involved in fatty acid synthesis (Lipe and Scd1), fatty acid oxidation (Cpt1a), triglyceride synthesis (Dgat1) and a lipid droplet-associated protein (Plin2) (78). Additionally, PPARγ ligands stimulate the expression of CD36, which is a scavenger receptor that facilitates the import of exogenous fatty acids (78). Therefore, it is feasible that Treg cells could use lipid stores by regulating lipolysis to provide FFAs for both catabolic and anabolic purposes. These data are consistent with the fact that Tregs preferentially utilize lipid oxidation for energy in contrast to effector T cells that primarily utilize glycolysis for energy generation (117).

In summary, Treg cells residing in adipose tissue are attuned to the local metabolic environment, which is associated with their unique metabolic profile, phenotype and function. Moreover, it is important to realize that some features of adipose Treg cell metabolism are generally similar to Treg cells residing in different microenvironments. For example, a key signaling pathway that controls Treg cell metabolism is regulated by the lipid phosphatase PTEN (118), which can be found in adipose tissue Treg cells. Treg induction has been shown to be significantly enhanced in naïve CD4^+^T cells in PTEN-overexpressing mice, which harbor Foxp3^+^Treg cells in adipose tissues (80). Mechanistically, Treg cell-specific deletion of PTEN leads to hyperglycolytic state, which destabilizes the Treg lineage via an effect on CD25 and Foxp3 expression (119). Therefore, these studies suggest that PTEN in Treg cells shifts their metabolic profile, which controls cellular function and cell fate decisions. Nonetheless, unraveling phenotype-specific signaling for determining the activation status and function of tissue-resident immune cells remains to be studied.

## Therapeutic Implications

Complex alterations in adipose immune cells offer therapeutic opportunities to control inflammatory reactions by disrupting inappropriate responses in the context of obesity and T2D. In principle, therapeutic benefits can be achieved by increasing the anti-inflammatory response and/or by reducing proinflammatory responses and promoting the non-immune function of local immune cells in obese adipose tissues.

The close coupling between the production of inflammatory mediators and the stages of disease progression provides clinical approaches to explore whether anti-inflammatory drugs could prevent obesity and T2D. For example, some anti-inflammatory treatments, such as anti-IL-1β, anti-TNF agents and salsalate, have been investigated for their potential to improve insulin resistance and prevent or ameliorate T2D (19). However, these functional improvements occurred differently; some improved insulin secretion (120) and some improved insulin sensitivity (121). This could reflect different target organs that are specifically associated with the immune response during the course of obesity and T2D.

Adoptive transfer of specific immune cells can also improve insulin resistance and T2D. In an attempt to promote beige adipocytes in obesity, IL-33-elicited ILC2s were transferred and led to increased uncoupling protein 1 (UCP1) beige adipocytes, a process that regulates caloric expenditure (13). Transferred ILC2 in treated wild-type mice was identified in white adipose tissue only and not in brown adipose tissue, mesenteric lymph nodes and lungs, indicating their selective accumulation in recipient mice (13). In the context of iNKT cell-based therapy for obese iNKT-deficient mice, this is also an exciting outcome because it suggested that adipose iNKT cells represent an important regulatory population, which induces an anti-inflammatory phenotype in macrophages and controls the number and suppressor function of Treg cells (10, 92). Thus, adoptive transfer of iNKT cells into obese mice decreased body fat, triglycerides, leptin, fatty liver, and improved insulin sensitivity (10). These studies highlight the potential of iNKT cell and ILC2-targeted adoptive transfer therapies in the management of obesity and its consequences.

Another promising therapeutic approach may be targeting transcription factors that specifically control the phenotype and function of adipose immune cells. It is well documented that PPARγ is critical in regulating inflammation by targeting adipose tissue Treg cells in obesity. As previously mentioned, injection of the PPARγ agonist pioglitazone into mice increased the number of adipose Treg cells and improved high-fat diet-associated syndrome. In addition to PPARγ, other transcription factors, such as IRF5, STAT3 and STAT4, are implicated in immune cells and can contribute to their function in obese tissues (103). In the future, the targeting of transcription factors will likely maintain a central role, mainly because transcription factors are always play a role in controlling the differentiation and function of immune cells and adipocytes.

## Conclusions

In recent years, a large number of studies in animal models have demonstrated the roles of adipose tissue immune cells in the regulation of inflammatory responses and glucose metabolism in the context of obesity and T2D. Although studies of adipose tissue immune cells in humans are limited, results are always consistent with those in animals (30, 62, 63, 70, 74). Additionally, most studies focused on visceral adipose tissue, because it was more metabolically active and its accumulation was associated with metabolism and insulin resistance (122). However, adipose tissue in different anatomical locations is also different in terms of molecular, cellular, anatomical features, and the capacity for fatty acid mobilization (123). Thus, much work is required to complete our understanding of adipose tissue immune cells in different anatomical locations.

A common feature of immune cells is difficult to summarize because both pathogenic and protective functions of a given immune cell (e.g., iNKT cells and γδ T cells) have been reported during disease development. This complexity is confounded by the fact that some immune cells can be modulated by shared signals, and an individual immune cell can influence a wide range of cell populations in specific microenvironments and disease contexts. Nevertheless, elevated levels of immunosuppressive immune cells such as ILC2s and Treg cells can contribute to protective effects in obesity and obesity-induced T2D. The current challenge is to elucidate how individual immune cells and ultimately the immune cell network contribute to disease development in the context of obesity. Therefore, the issue is whether targeting select immune cell populations in the presence of the immune system can facilitate a change in their function and coordinate local immune responses, further contributing to the prevention of diseases.

The identification of previously unrecognized immune cell subpopulations that reside in adipose tissues adds greater complexity to understanding the immune cell network in the context of obesity. It is also important to further probe other principal immune cell subtypes, to obtain their unique signatures and to understand how they are dysregulated in the context of obesity and T2D. However, adipose tissue is not the sole site for immune cell dysregulation and metabolic inflammation during obesity, which also occurs in other tissues, such as the gut (124, 125), liver (126), and pancreatic islets (127). The systemic impact of metabolic inflammation and immune cells on the development of insulin resistance needs to be critically considered in determining physiological and pathological outcomes.

## Author Contributions

All authors listed have made a substantial, direct and intellectual contribution to the work, and approved it for publication.

### Conflict of Interest Statement

The authors declare that the research was conducted in the absence of any commercial or financial relationships that could be construed as a potential conflict of interest.

## Abbreviations

ACC: acetyl-CoA carboxylase
AMPK: AMP kinase
Breg cell: regulatory B cell
CCL: C-C motif chemokine ligand
cDCs: conventional DCs
CHOP: C/EBP homologous protein
DC: dendritic cell
ER: endoplasmic reticulum
FFA: free fatty acids
GM-CSF: granulocyte-macrophage colony stimulating factor
GPS2: G protein pathway suppressor 2
HIF: hypoxia-inducible factor
Id2: DNA binding 2
IKK: IκB kinase
IL: interleukin
ILCs: innate lymphoid cells
iNKT: invariant natural killer T cells
IRE1: inositol-requiring enzyme 1
Irf5: interferon regulatory factor 5
JNK: c-Jun N-terminal kinase
MAIT: mucosal-associated invariant T cells
MEKK3: mitogen-activated kinase kinase 3
mTORC1: mammalian target of rapamycin complex 1
NF-κB: nuclear factor-κB
pDCs: plasmacytoid DCs
PGC1: PPARγ coactivator−1β
PPAR: peroxisome proliferators-activated receptor
T2D: type 2 diabetes
TCF-7: transcription factor 7
TNF: tumor necrosis factor
Th1: T helper 1
TZD: thiazolidinedione
UCP1: uncoupling protein 1.
